# The Role of Hs-CRP, D-Dimer and Fibrinogen in Differentiating Etiological Subtypes of Ischemic Stroke

**DOI:** 10.1371/journal.pone.0118301

**Published:** 2015-02-13

**Authors:** Li-Bin Liu, Mu Li, Wen-Yan Zhuo, Yu-Sheng Zhang, An-Ding Xu

**Affiliations:** 1 Department of Neurology, the First Affiliated Hospital of Jinan University, Guangzhou, China; 2 Department of Neurology, Zhuhai Hospital of Jinan University, Zhuhai, China; St Michael's Hospital, University of Toronto, CANADA

## Abstract

The aim of this study was to evaluate the diagnostic value of the serum biochemical markers high-sensitivity C-reactive protein (hs-CRP), D-dimer (DD) and fibrinogen (Fg) in differentiating etiological subtypes of ischemic stroke. This study was a retrospective case-only study, consecutively including patients with acute ischemic stroke. All patients were classified into subtypes using the TOAST classification system. A total of 317 patients were evaluated. Hs-CRP and DD levels were significantly different among the subtypes and were the highest in CE, followed by LAA and SAA; no significant difference between the subtypes was found for Fg. Hs-CRP > 6.96 mg/L was classified as the CE subtype, with a sensitivity of 41% and a specificity of 74%; DD > 791.30 ng/mL was classified as CE, with a sensitivity of 58% and a specificity of 78%. The combination of hs-CRP and DD classification as CE yielded a sensitivity of 65% and a specificity of 91%. DD > 791.30 ng/mL was considered an independent predictive factor of CE. Hs-CRP and DD could be useful for identifying the etiological subtypes of acute ischemic stroke, especially for predicting CE. The diagnostic value of DD was higher than that of hs-CRP.

## Introduction

Ischemic stroke is not a single disease but a group of diseases with many different etiologies. Therefore, the etiological classification of ischemic stroke is important for choosing the appropriate patient care and secondary prevention programs.

However, precise etiological classification depends heavily on the results of auxiliary examination, such as cranial imaging, echocardiography, carotid vascular ultrasound and transesophageal echocardiography. Most of these examinations are difficult to complete at the early onset of a stroke. These factors plus a lack of appropriate equipment in the primary hospital can make timely and accurate classification impossible. Moreover, cardiogenic cerebral embolism has a lower diagnostic rate and a higher missed diagnosis rate in Chinese clinical practice than abroad. This disparity can largely be attributed to this dearth of targeted assessments.

Little published evidence is available on serum biochemical markers for the etiological classification of ischemic stroke. The current study aimed to observe the serum levels of high-sensitivity C-reactive protein (hs-CRP), D-dimer (DD) and fibrinogen (Fg), which can all be obtained quickly in various subtypes of TOAST etiological classifications, to provide clinical biochemical parameters and a basis for the etiological classification of patients with acute ischemic stroke. These data will enable earlier classification, guide targeted examinations, and inform rational intervention treatment for ischemic stroke.

## Subjects and Methods

### Subjects

This retrospective, case-only study was approved by the medical ethics committee of the First Affiliated Hospital of Jinan University, Guangzhou. Written informed consent was not obtained and the patients’ records/information were anonymized and de-identified prior to analysis. We consecutively enrolled patients with acute ischemic stroke hospitalized in the Department of Neurology at the First Affiliated Hospital of Jinan University from January 2009 to December 2010. All patients had been discharged, and were identified the etiological subtypes before hospital discharge. They were divided into 5 subtypes groups. Then we compared the levels of three kinds of serum biochemical markers among these groups.

**Inclusion Criteria**. Patients were chosen (1) whose cases conformed to the diagnostic criteria of the 2005 China cerebrovascular disease prevention and treatment guidelines for ischemic stroke; (2) whose time of stroke onset was not more than 7 days prior to inclusion; and (3) who had no history of ischemic stroke within the previous 6 months.

**Exclusion Criteria**. Patients were excluded if they (1) were below 18 years of age; (2) had a diagnosis of hemorrhagic stroke and transient ischemic attack; (3) had systemic infections, autoimmune diseases, malignancies, serious heart, liver or kidney diseases, blood disorders, or other systemic thromboembolic pathologies; (4) had experienced a myocardial infarction, acute trauma, or surgery within the previous three months; (5) took long-term antithrombotic drugs (including anti-platelet drugs, anticoagulants, fibrinolytics, statins lipid-lowering drugs, inflammation inhibitors, immunosuppressive agents or hormones); or (6) underwent thrombolytic therapy after admission.

### Methods

**Collection of General Data**. All of the patients’ data were collected from their medical records, including all of the demographic data (e.g., gender and age), the history of their present illness, their personal and family medical history, the relevant imaging data (i.e., cranial MR and CT, echocardiography, and dynamic electrocardiogram), laboratory tests (i.e., routine blood tests, blood lipids, blood glucose, and glycosylated hemoglobin), and other examination results helpful for clarifying the cause of the disease.

**Specimen Detection Method**. Hs-CRP, DD and Fg were all measured in our hospital laboratory. The venous blood was collected, anticoagulated using sodium citrate, and then centrifuged at 3000 r/min for 10 minutes for the isolation of serum.

To determine hs-CRP and DD levels in the sera, 4 mL of fasting cubital venous blood was collected from each patient in the morning after the day they were admitted to the hospital. Hs-CRP levels were measured with reagents produced by Germany Siemens Healthcare Diagnostics Products Co., Ltd. and particle-reinforced immune scattering nephelometry using a BN system. DD levels were measured with a kit and immunoturbidimetry using an automatic biochemical detector which were both produced by Beijing Leadman Biochemistry Co., Ltd.

Additionally, 3 mL of cubital venous blood was collected from each patient within 3 hours of hospital admission, and the Fg levels were determined using the coagulation method with an SF-8000 automatic coagulation analyzer produced by Beijing Saikexide Technology Development Co., Ltd. and its special hemagglutination reagents.

**Classification of Ischemic Stroke**. All of the study participants were classified using the patients’ medical records, and the professionals in our Stroke Unit discussed and classified the ischemic stroke cases into subtypes according to the TOAST etiology classification system [1], divided into five categories: large-artery atherosclerosis (LAA), cardioembolism (CE), small-artery occlusion (SAA), stroke of other determined etiology (SOE), and stroke of undetermined etiology (SUE).

**Statistical Analysis**. The SPSS13.0 statistical software was used to analyze all of the data. First, all of the patients’ general data were analyzed; the chi-square test was used for the counted data, and an analysis of variance or a rank sum test was used to compare the measured data. The levels of the three biochemical markers in each subtype that had a normal distribution are expressed as the means ± standard deviation (*X̅*±*s*). The marker levels with skewed distributions are expressed as the interval of the median and quartile. The means were compared using t-tests between the two groups and multi-sample mean analysis of variance among multiple groups; due to the heterogeneity of the variance among the different groups, the groups with non-normal distributions were compared with rank sum tests for two-sample or multi-sample comparisons. A receiver operator characteristic (ROC) curve was configured to calculate the sensitivity and specificity of the biochemical markers’ cutoff values for predicting a specific stroke etiology. A combined test was used to evaluate the use of both biomarkers to predict the stroke subtype. The multivariate analyses used logistic regression analysis.

## Results

### Baseline Data

There were a total of 317 patients studied (Table 1), including 202 cases of males (63.72%) and 115 cases of females (36.28%), with an average age of 64.91 ± 12.33 years. The SOE cases were not included in the statistical analysis due to their small number. As shown in Table 1, no statistically significant difference was observed in the presence of diabetes, LDL-C levels, the numbers of smokers, or alcohol consumption among the various subtypes. The proportion of patients with hypertension was higher in the LAA group, and the proportion of females was higher in the CE group.

**Table 1:** Baseline data of various stroke subtypes.

Baseline parameters	LAA	CE	SAA	SOE	SUE	Total	*P* Value
Number	162 (51.10%)	37 (11.67%)	62 (19.56%)	3 (0.95%)	53 (16.72%)	317	0.000
Males	108 (66.67%)	13 (35.14%)	42 (67.74%)	2 (66.67%)	37 (69.81%)	202 (63.72%)	0.002
Age, years (mean±SD)	65.70±10.64	68.73±14.24	62.44±11.97	39.33±22.19	64.17±13.72	64.91±12.33	0.000
Hypertension	146 (90.12%)	22 (59.46%)	54 (87.10%)	1 (33.33%)	44 (83.02%)	267 (84.23%)	0.000
Diabetes	44 (27.16%)	6 (16.22%)	15 (24.19%)	0	13 (24.53%)	78 (24.61%)	0.581
LDL-C (mmol/L)	2.97±0.73	2.57±0.81	3.01±0.78	1.92±0.62	2.80±0.79	2.89±0.77	0.463
Smoking	64 (39.51%)	9 (24.32%)	26 (41.94%)	1 (33.33%)	19 (35.85%)	119 (37.54%)	0.308
Drinking	24 (14.81%)	2 (5.41%)	7 (11.29%)	0	8 (15.09%)	41 (12.93%)	0.438

### Specific Results of Classification

All patients were classified into five subtypes, including 162 cases (51.10%) of LAA, 37 cases (11.67%) of CE, 62 cases (19.56%) of SAA, 3 cases (0.95%) of SOE, and 53 cases (16.72%) of SUE (Table 1). The 37 CE cases included 28 cases of patients with atrial fibrillation, 5 cases of patients with sick sinus syndrome, one patient who underwent mitral valve replacement surgery (without atrial fibrillation), one patient with a patent foramen ovale, one case of dilated cardiomyopathy and one case of mitral valve prolapse. Polycythemia vera, arteritis, arteriovenous malformations and arteriovenous fistulae were observed in the three cases of SOE.

### Biochemical Marker Distribution in the Five Subtypes

The specific results are shown in Table 2. The SOE group was not included in the statistical analysis because of the small number of cases. The SUE group included some cases with both LAA and CE and some cases in which the etiology could not be determined, so SUE was also excluded from the statistical analysis. The Kruskal-Wallis H test was used to compare the levels of hs-CRP and DD among the remaining stroke subtypes, revealing that the levels of hs-CRP were different in each group (*χ2* = 13.38, *P* = 0.001); the levels were highest in the CE group, followed by the LAA group, and the levels were the lowest in the SAA group. Similarly, the DD levels were also different between the subtypes (*χ2* = 26.14 and *P* = 0.000); they were the highest in the CE patients, followed by the LAA group, and the levels were lowest in the SAA group. Finally, a multi-sample mean analysis of variance and Student-Newman-Keuls test, *F* = 0.881 and *P* = 0.416 (> 0.05) showed that the level of Fg was not significantly different between the subtypes.

**Table 2:** Distribution of three biochemical markers in various subtypes.

	LAA	CE	SAA	SUE	*P* Value
hs-CRP (mg/L)	3.22 (1.54–7.88)	3.59 (1.11–9.85)	1.88 (0.56–4.46)	2.86 (0.98–7.04)	0.001
DD (ng/mL)	439.26 (260.94–783.14)	945.24 (388.91–1816.04)	294.16 (167.53–455.79)	324.68 (225.21–684.00)	0.000
Fg (g/L)	3.11±0.75	3.03±0.89	2.96±0.77	2.94±0.78	0.416

### ROC curve of hs-CRP and DD

To evaluate the diagnostic value of hs-CRP and DD serum levels to predict CE, the SPSS13.0 software was used to obtain ROC curves and determine the cut-off points. The area under the ROC curve (Az) of CRP was 0.545 (generally, Az > 0.5 is considered diagnostic, whereas Az ≤ 0.7 indicates a lower diagnostic value; 0.7 < Az ≤ 0.9 indicates a moderate diagnostic value; Az > 0.9 indicates a high diagnostic value), indicating that serum hs-CRP is not useful for diagnosing CE. Those with cut-off points of 6.96, that is hs-CRP > 6.96 mg/L, were classified as CE with a sensitivity of 41% and a specificity of 74%. The positive predictive value was 18% and the negative predictive value was 90%. The area under the ROC curve of DD was 0.712, indicating that serum DD was moderately diagnostic for CE. Those with cut-off points of 791.30, that is DD > 791.30 ng/mL, were classified as CE with a sensitivity of 58% and a specificity of 78%. The positive predictive value was 26% and the negative predictive value was 93%.

### Hs-CRP Combined with DD in the Diagnosis of CE

As shown above, the lone determination of hs-CRP > 6.96 mg/L or DD> 791.30 ng/mL to determine a diagnosis of CE generated poor results in both sensitivity and specificity. Therefore, we wondered whether the hs-CRP and DD criteria could be combined to improve the sensitivity and specificity of the test, thereby improving the efficiency of diagnosis. If a diagnosis of CE included levels higher than the cut-off point for either hs-CRP or DD, the two diagnostic methods would be parallel diagnostic tests, which could improve the sensitivity of the test but would decrease the specificity. The combined evaluation indicators shown in Table 3 were calculated as follows: sensitivity = 22/34 × 100% = 65%, specificity = 132/215 × 100% = 61%, positive predictive value = 22/105 × 100% = 21%, and negative predictive value = 132/144 × 100% = 92%.

**Table 3:** Evaluation of hs-CRP and DD in combined diagnosis of CE (parallel diagnostic tests).

hs-CRP and DD Value	Number of CE	Number of non-CE	Total
hs-CRP>6.96 mg/L or/and DD>791.30 ng/mL	22	83	105
hs-CRP<6.96 mg/L and DD<791.30 ng/mL	12	132	144
Total	34	215	249

If a diagnosis of CE was based on both hs-CRP and DD being higher than the cut-off points, then the two diagnostic methods could be considered a series of diagnostic tests, which would improve the specificity of the test but decrease the sensitivity. The combined evaluation indicators shown in Table 4 were calculated as follows: sensitivity = 12/34 × 100% = 35%, specificity = 195/215 × 100% = 91%, positive predictive value = 12/32 × 100% = 38%, and negative predictive value = 195/217 × 100% = 90%.

**Table 4:** Evaluation of hs-CRP and DD in combined diagnosis of CE (series of diagnostic tests).

hs-CRP and DD Value	Number of CE	Number of non-CE	Total
hs-CRP>6.96 mg/L and DD>791.30 ng/mL	12	20	32
hs-CRP<6.96 mg/L or/and DD<791.30 ng/mL	22	195	217
Total	34	215	249

### Logistic Regression Analysis of Cardioembolism

As mentioned above, the levels of hs-CRP and DD were the highest in the CE subtype; hs-CRP > 6.96 mg/L and DD > 791.30 ng/mL were the cut-off points for the diagnosis of CE. To evaluate the risk factors for CE, hs-CRP > 6.96 mg/L, DD > 791.30 ng/mL, hypertension, advanced age (≥ 65 years) and male were entered into the logistic regression model. By multivariate logistic regression analysis, the variables were screened using the forward method. The inspection level α = 0.10 of the variables was introduced. The data were adjusted for confounding factors such as gender, age, and hypertension. Only DD > 791.30 ng/mL was an independent predictor of CE (*OR* 6.825, 95% *CI*: 2.955 ∼ 15.766, *P* = 0.000).

## Discussion

### The Relationship between Hs-CRP and Ischemic Stroke Subtypes

Hs-CRP is a typical acute-phase protein, widely present in the serum and other body fluids. The median value of plasma levels of hs-CRP in healthy people is approximately 1 mg/L and significantly increases 100 times or more in cases of tissue injury, infection or inflammation [2]. A number of studies have shown that hs-CRP is significantly increased in patients with ischemic stroke [3–5], which was related to the severity of ischemic stroke.

Our current study demonstrated that among the TOAST subtypes, the level of hs-CRP was the highest in CE patients followed by LAA cases and was the lowest in the SAA group, consistent with the results of previous studies [3,5–7]. Several reasons could explain the high hs-CRP levels in CE cases. First, hs-CRP levels are generally increased in heart disease [8] and are particularly higher in patients with atrial fibrillation and heart failure [9], the most common sources of cardioembolism. Interestingly, although many studies have shown that hs-CRP levels are related to aortic atherosclerosis, more recent studies have shown no significant correlation between the two factors [10,11]. Second, the increased range of hs-CRP levels was related to the degree of brain tissue damage [12,13]. CE often leads to multi-site and extensive areas of cerebral infarction, resulting in a higher level of hs-CRP. In contrast, the area of cerebral infarction in patients with small artery disease was relatively smaller, with less inflammation; therefore, the hs-CRP level was lower.

### The Relationship of DD to Ischemic Stroke Subtypes

DD is a terminal degradation product produced by plasmin hydrolysis after cross-linking of fibrin monomer and thrombin activator XIII cross-linked (i.e., cross-linked fibrin) [14] and is one of the specific fibrinolysis markers. The level of DD is very low in the peripheral blood of healthy people, but significantly increases during the thrombosis and secondary fibrinolysis processes but not in primary fibrinolysis. The reference value of plasma DD measured using ELISA was below 200 ng/mL. A number of studies have confirmed that the plasma level of DD increases in patients with acute ischemic stroke compared with healthy people [15,16].

In recent years, there have been an increase number of studies on DD and the etiological subtypes of stroke. This study showed that the plasma levels of DD in the CE group were significantly higher than in the other subtypes, with the lowest in SAA, which is consistent with many previously published results [17–21]. When ischemic stroke occurs, the increased activity of the coagulation system reflects the mechanism of thrombosis and vascular occlusion. In fact, from the specific mechanism, atrial or ventricular thrombus formation mainly occurs due to stillness or stagnation of local blood. The local level of clotting factor increases to activate the clotting process, thereafter resulting in the formation of a fibrin-rich clot; the process is very similar in venous thrombosis. In contrast, arterial thrombosis is rich in platelets, and fibrin formation is secondary to the activation of platelets. Therefore, more DD is produced by the degradation of a cardioembolism after the activation and secondary onset of fibrinolysis. In the traditional view, SAA is mainly caused by lipid hyaline change in perforating arteries due to hypertension or diabetes. In recent years, this model was amended such that SAA was also partly caused by atherosclerotic thrombotic occlusion of the perforating artery. However, the secondary fibrinolysis was often lower than the atherosclerotic thrombotic occlusion of the main artery. Therefore, the level of DD was the highest in CE cases and the lowest in SAA cases.

### The Relationship between Fg and Ischemic Stroke Subtypes

There have been few studies on the relationship between the level of Fg and the subtypes of ischemic stroke, and those have generated inconsistent results. Turaj et al. [22] showed that the level of Fg was the highest in LAA. Vibo et al. [23] found that Fg was not significantly different among various subtypes, which was consistent with this study. Whether a high plasma Fg level is a risk factor for stroke or is the body’s reaction after a stroke is unclear. Several large-sample prospective studies [24–26] have shown that people with high levels of basic Fg have a higher risk of stroke than those with low levels. Further, Fg was an independent predictor of ischemic stroke. Another study [27] showed that Fg after stroke was not significantly different from the control group not suffering from stroke. In addition, Carter et al. [28] found that the relationship between Fg and cerebrovascular disease might depend on gender. In that study, the male subjects had increased levels of Fg and the female subjects exhibited changes in Fg function. As reported, the plasma level of Fg was not high, but in the case of structural abnormalities, the reactivity of Fg was increased, which might also lead to thrombosis and other pathological changes. As a coagulation factor, in cases of local thrombosis, a very small amount of Fg would be converted into fibrin by thrombin and then the consumed Fg might be regenerated by the liver. Fg in patients with ischemic stroke might be slightly elevated or not elevated, and its level among various subtypes of stroke might be not significantly different. However, as an acute-phase protein, the elevated level of Fg has also been related to the volume of brain infarct. Altogether, the existing data support our finding that Fg is the lowest in SAA.

### The Relationship between hs-CRP, DD and CE

Our study shows that the levels of hs-CRP and DD are higher in CE patients than in the other subtypes and that this difference is statistically significant. The respective cut-off point obtained from the ROC curve was 6.96 for hs-CRP and 791.30 for DD. The significance of the cut-off point is that the standard of hs-CRP > 6.96 mg/L could be used to predict an ischemic stroke as the CE type but with a sensitivity of only 41% and a specificity of 74%; the positive predictive value was 18%, meaning that those with the CE type stroke accounted for 18% of all patients with hs-CRP > 6.96 mg/L. The negative predictive value was 90%, which means that among all of the patients with hs-CRP < 6.96 mg/L, 90% of them could be classified as the non-CE type. In fact, the negative predictive value was the most valuable evaluation indicator. For example, the negative predictive value of DD was 93%, indicating that among all of the patients with DD < 791.30 ng/mL, 93% of them could be categorized as non-CE type. Furthermore, the area under the ROC curve demonstrates that the diagnostic value of DD was higher than hs-CRP for CE.

A combined test method can be used to improve the accuracy of a single diagnostic test. Combined tests can be composed of parallel and serial diagnostic tests. The former would improve the combined sensitivity, while the latter would improve the combined specificity. If a diagnosis of CE was based on either hs-CRP or DD being higher than the cut-off point, the two diagnostic methods would be considered parallel diagnostic tests. The combined evaluation indicators were as follows: sensitivity = 65%, specificity = 61%, positive predictive value = 21%, and negative predictive value = 92%. If a diagnosis of CE was based on both hs-CRP and DD being higher than the cut-off point, the two diagnostic methods would be considered a series of diagnostic tests. The combined evaluation indicators were as follows: sensitivity = 35%, specificity = 91%, positive predictive value = 38%, negative predictive value = 90%.

Moreover, the multivariate logistic regression analysis showed that DD> 791.30 ng/mL was an independent predictor of CE (*OR* 6.825, 95% *CI*: 2.955 ∼ 15.766, *P* = 0.000).

Several studies have probed DD to determine the cut-off value for predicting CE. Ageno et al. [17] found that the most appropriate cut-off value for the prediction of CE was 2000 ng/mL, with a specificity of 93.2% and a sensitivity of 59.3%. The cut-off value for predicting SAA was 540 ng/mL, with a specificity of 96.2% and a sensitivity of 61.3%. Montaner et al. [19] showed that the diagnostic sensitivity of DD > 960 ng/mL for CE was 56%, with a specificity of 64%, a positive predictive value of 46%, and a negative predictive value of 73%. The diagnostic sensitivity of BNP (brain natriuretic peptide) > 76 pg/mL for CE was 72%, with a specificity of 68%. A combined diagnostic method composed of DD and BNP also improved the sensitivity (87%) and specificity (85%). Isenegger et al. [20] showed in 2010 that CE could be excluded in patients with DD levels below 300 ng/mL within 6 hours after the stroke onset, with a sensitivity of 100% and a specificity of 52%.

The current study shows that DD and hs-CRP are potential markers of CE. These biochemical markers play specific roles in the diagnosis of CE and can assist in the early etiological classification of ischemic stroke, guiding the development of targeted assessments to improve the diagnostic yield of CE cases and accelerating the administration of appropriate treatment and secondary prevention programs. The combined evaluation of serum biochemical markers has the potential for improved sensitivity and specificity, thereby improving the diagnosis of stroke subtypes. Therefore, DD and hs-CRP might be used as screening indicators for the exclusion of CE early in the onset of ischemic stroke. Finding more diagnostic biochemical markers that work similarly to hs-CRP and DD for CE will allow a more accurate method of classifying stroke etiological subtypes and could be combined for more accurate diagnosis of CE.

## Conclusions

Hs-CRP and DD could be useful for identifying the etiological subtypes of acute ischemic stroke, especially for predicting CE. The diagnostic value of DD was higher than that of hs-CRP. Using a combination of hs-CRP and DD may be a feasible strategy for improving the diagnosis accuracy of CE in the acute phase.

## References

[1] Adams HP Jr, Bendixen BH, Kappelle LJ, Biller J, Love BB, Gordon DL, et al Classification of subtype of acute ischemic stroke. Definitions for use in a multicenter clinical trial. TOAST. Trial of Org 10172 in Acute Stroke Treatment. Stroke. 1993;24(1): 35–41. 7678184 10.1161/01.str.24.1.35

[2] Cao JJ, Thach C, Manolio TA, Psaty BM, Kuller LH, Chaves PH, et al C-reactive protein, carotid intima-media thickness, and incidence of ischemic stroke in the elderly: the Cardiovascular Health Study. Circulation. 2003;108(2): 166–170. 12821545 10.1161/01.CIR.0000079160.07364.6A

[3] Eikelboom JW, Hankey GJ, Baker RI, McQuillan A, Thom J, Staton J, et al C-reactive protein in ischemic stroke and its etiologic subtypes. J Stroke Cerebrovasc Dis. 2003;12(2): 74–81. 17903908 10.1053/jscd.2003.16

[4] Pandey A, Shrivastava AK, Saxena K. Neuron specific enolase and c-reactive protein levels in stroke and its subtypes: correlation with degree of disability. Neurochem Res. 2014;39(8): 1426–1432. 10.1007/s11064-014-1328-9 24838548

[5] Luo Y, Wang Z, Li J, Xu Y. Serum CRP concentrations and severity of ischemic stroke subtypes. Can J Neurol Sci. 2012;39(1): 69–73. 22384498 10.1017/s0317167100012713

[6] Terruzzi A, Valente L, Mariani R, Moschini L, Camerlingo M. C-reactive protein and aetiological subtypes of cerebral infarction. Neurol Sci. 2008;29(4): 245–249. 10.1007/s10072-008-0975-5 18810599

[7] Ladenvall C, Jood K, Blomstrand C, Nilsson S, Jern C, Ladenvall P, et al Serum C-reactive protein concentration and genotype in relation to ischemic stroke subtype. Stroke. 2006;37(8): 2018–2023. 16809555 10.1161/01.STR.0000231872.86071.68

[8] Habib SS, Masri A AAI. Relationship of high sensitivity C-reactive protein with presence and severity of coronary artery disease. Pak J Med Sci. 2013;29(6): 1425–1429. 24550967 10.12669/pjms.296.3302 PMC3905368

[9] Anderson JL, Allen Maycock CA, Lappe DL, Crandall BG, Horne BD, Bair TL, et al Frequency of elevation of C-reactive protein in atrial fibrillation. Am J Cardiol. 2004;94(10): 1255–1259. 15541240 10.1016/j.amjcard.2004.07.108

[10] Chapman CM, Beilby JP, McQuillan BM, Thompson PL, Hung J. Monocyte count, but not C-reactive protein or interleukin-6, is an independent risk marker for subclinical carotid atherosclerosis. Stroke. 2004;35(7): 1619–1624. 15155967 10.1161/01.STR.0000130857.19423.ad

[11] Lorenz MW, Karbstein P, Markus HS, Sitzer M. High-sensitivity C-reactive protein is not associated with carotid intima-media progression: the carotid atherosclerosis progression study. Stroke. 2007;38(6): 1774–1779. 17446427 10.1161/STROKEAHA.106.476135

[12] Kara H, Akinci M, Degirmenci S, Bayir A, Ak A, Nayman A, et al High-sensitivity C-reactive protein, lipoprotein-related phospholipase A2, and acute ischemic stroke. Neuropsychiatr Dis Treat. 2014;10: 1451–1457. 10.2147/NDT.S67665 25125979 PMC4130710

[13] Beer C, Blacker D, Hankey GJ, Puddey IB. Association of clinical and aetiologic subtype of acute ischaemic stroke with inflammation, oxidative stress and vascular function: a cross-sectional observational study. Med Sci Monit. 2011;17(9): CR467–473. 21873941 10.12659/MSM.881931 PMC3560506

[14] Marder VJ, Francis CW. Plasmin degradation of cross-linked fibrin. Ann N Y Acad Sci. 1983;408: 397–406. 6223558 10.1111/j.1749-6632.1983.tb23260.x

[15] Tombul T, Atbas C, Anlar O. Hemostatic markers and platelet aggregation factors as predictive markers for type of stroke and neurological disability following cerebral infarction. J Clin Neurosci. 2005;12(4): 429–434. 15925775 10.1016/j.jocn.2004.06.013

[16] Matsumoto M, Sakaguchi M, Okazaki S, Furukado S, Tagaya M, Etani H, et al Relationship between plasma (D)-dimer level and cerebral infarction volume in patients with nonvalvular atrial fibrillation. Cerebrovasc Dis. 2013;35(1): 64–72. 10.1159/000345336 23428999

[17] Ageno W, Finazzi S, Steidl L, Biotti MG, Mera V, Melzi D’Eril G, et al Plasma measurement of D-dimer levels for the early diagnosis of ischemic stroke subtypes. Arch Intern Med. 2002;162(22): 2589–2593. 12456231 10.1001/archinte.162.22.2589

[18] Koch HJ, Horn M, Bogdahn U, Ickenstein GW. The relationship between plasma D-dimer concentrations and acute ischemic stroke subtypes. J Stroke Cerebrovasc Dis. 2005;14(2): 75–79. 17904004 10.1016/j.jstrokecerebrovasdis.2004.12.002

[19] Montaner J, Perea-Gainza M, Delgado P, Ribó M, Chacón P, Rosell A, et al Etiologic diagnosis of ischemic stroke subtypes with plasma biomarkers. Stroke. 2008;39(8): 2280–2287. 10.1161/STROKEAHA.107.505354 18535284

[20] Isenegger J, Meier N, Lämmle B, Alberio L, Fischer U, Nedeltchev K, et al D-dimers predict stroke subtype when assessed early. Cerebrovasc Dis. 2010;29(1): 82–86. 10.1159/000256652 19907168

[21] Zi WJ, Shuai J. Plasma D-dimer levels are associated with stroke subtypes and infarction volume in patients with acute ischemic stroke. PLoS One. 2014;9(1): e86465 10.1371/journal.pone.0086465 24466108 PMC3896474

[22] Turaj W, Słowik A, Pułyk R, Adamski M, Szczudlik A. Comparison of plasma concentrations of fibrinogen in patients with ischemic stroke due to large vessel disease and small vessel disease. Neurol Neurochir Pol. 2006;40(4): 297–301. 16967351

[23] Vibo R, Kõrv J, Roose M, Kampus P, Muda P, Zilmer K, et al Acute phase proteins and oxidised low-density lipoprotein in association with ischemic stroke subtype, severity and outcome. Free Radic Res. 2007;41(3): 282–287. 17364956 10.1080/10715760601083235

[24] Eidelman RS, Hennekens CH. Fibrinogen: a predictor of stroke and marker of atherosclerosis. Eur Heart J. 2003;24(6): 499–500. 12643881 10.1016/s0195-668x(02)00810-2

[25] Siegerink B, Rosendaal FR, Algra A. Genetic variation in fibrinogen; its relationship to fibrinogen levels and the risk of myocardial infarction and ischemic stroke. J Thromb Haemost. 2009;7(3): 385–390. 10.1111/j.1538-7836.2008.03266.x 19143925

[26] Chuang SY, Bai CH, Chen WH, Lien LM, Pan WH. Fibrinogen independently predicts the development of ischemic stroke in a Taiwanese population: CVDFACTS study. Stroke. 2009;40(5): 1578–1584. 10.1161/STROKEAHA.108.540492 19286580

[27] Giannopoulos S, Kosmidou M, Hatzitolios AI, Savopoulos CG, Ziakas A, Karamouzis M. Measurements of endothelin-1, C-reactive protein and fibrinogen plasma levels in patients with acute ischemic stroke. Neurol Res. 2008;30(7): 727–730. 10.1179/174313208X297904 18489822

[28] Carter AM, Catto AJ, Bamford JM, Grant PJ. Gender-specific associations of the fibrinogen Bβ448 polymorphism, fibrinogen levels, and acute cerebrovascular disease. Arterioscler Thromb Vasc Biol. 1997;17(3): 589–594. 9102181 10.1161/01.atv.17.3.589

